# Experiences of internationally qualified nurses in adapting to the Australian healthcare system: A scoping review

**DOI:** 10.1016/j.ijnsa.2025.100399

**Published:** 2025-08-05

**Authors:** Ferry Efendi, Rifky Octavia Pradipta, Makhfudli Makhfudli, Lisa McKenna, Grace Solely Houghty, Fitri Kurnia Rahayu, Fildzah Cindra Yunita

**Affiliations:** a Department of Advance Nursing, Faculty of Nursing, Universitas Airlangga, Surabaya, Indonesia; b School of Nursing and Midwifery, La Trobe University, Melbourne, Australia; c Research Center in Advancing Community Healthcare (REACH), Universitas Airlangga, Surabaya, Indonesia; d Department of Fundamental Nursing, Faculty of Nursing, Universitas Airlangga, Surabaya, Indonesia; e Universitas Pelita Harapan, Jakarta, Indonesia; f Faculty of Public Health, Universitas Airlangga, Surabaya, Indonesia; g Research Group for Health and Wellbeing of Women and Children, Faculty of Public Health, Universitas Airlangga, Indonesia

**Keywords:** Adaptation, Internationally qualified nurse, Nurse migration, Integration, Foreign Workforce, Global Migration

## Abstract

**Background:**

Internationally qualified nurses commonly face challenges related to language barriers, cultural adaptation, and recognition of prior professional skills.

**Purpose:**

We aimed to map and synthesise the literature about the experiences of Internationally qualified nurses transitioning into the Australian healthcare system and to identify key challenges and gaps in supporting their integration and professional development.

**Methods:**

A scoping review was conducted using a comprehensive search across five databases (Ovid MEDLINE, EBSCO CINAHL, Embase, Scopus, and Web of Science Core Collections), covering the literature from January 2014 to August 2024. Two researchers screened, selected relevant, and performed data charting, independently. The Patterns, Advances, Gaps, Evidence for practice, and Research recommendations framework was implemented to collate, summarise, and report the finding, while the review’s report was formulated per the PRISMA-ScR guideline.

**Results:**

Fifteen studies were deemed suitable and synthesised from which we revealed that internationally qualified nurses underwent complex and multifaceted transitions. They faced language barriers, challenges in cultural adaptation, inadequate recognition of professional skills, and inconsistent access to support resources. Despite these hurdles, they demonstrated remarkable resilience. Language and communication challenges, particularly among nurses from non-English-speaking backgrounds, were common and impacted job satisfaction and integration. Meanwhile, discrimination and cultural biases were also reported, which contributed to feelings of isolation. Finally, orientation programs and mentorship proved beneficial support; however, inconsistencies in support availability remains a key gap.

**Conclusions:**

Internationally qualified nurses’ experiences reflected the need for tailored language support, standardised skill recognition, anti-discrimination policies, and consistent orientation resources to enhance integration. Addressing these gaps could not only improve internationally qualified nurses’ job satisfaction, retention, and overall professional development within the Australian healthcare system but also contribute to a more diverse and inclusive healthcare workforce.

**What is already known about the topic**

Internationally qualified nurses play a vital role in addressing workforce shortages within the Australian healthcare system.

There is growing concern over lack of consistent support systems, such as orientation and mentorship programs, for facilitating the integration of internationally qualified nurses.

**What this paper adds**

Internationally qualified nurses experienced professional dissonance, cultural isolation, and underutilisation of skills during transition into the Australian healthcare system, demonstrating resilience and adaptability.

We identified critical gaps in institutional support, including inconsistent recognition of foreign qualifications and limited access to structured orientation programs.

We have offered actionable recommendations for practice and policy, including tailored language support, anti-discrimination measures, and standardized skill validation pathways.

Alt-text: Unlabelled box

## 1 Introduction

Migration of internationally qualified nurses has become a critical component of the global healthcare workforce, addressing labour shortages in many countries (World Health Organization, 2020), including Australia. Internationally qualified nurses are pivotal in maintaining the stability of Australia's healthcare system, contributing to diverse care settings, from hospitals to aged care facilities (Smith et al., 2022). In the 2023–2024 financial year, Australia experienced a significant influx of Internationally qualified nurses, with 16,622 registering to practice—a threefold increase compared to the 5610 registrations in 2018–2019 (Butler, 2025). This surge reflects the country's growing reliance on internationally qualified nurses to address healthcare workforce shortages. While their presence is essential, the transition into the Australian healthcare system often poses significant challenges, impacting job satisfaction, professional identity, and retention (Dywili et al., 2021; Kurup et al., 2024). Despite growing reliance on internationally qualified nurses, little is known about the multifaceted experiences they face in transitioning or adapting to the Australian context, which hinders development of targeted policies and support mechanisms. In this review, ‘transition’ refers to the multifaceted process of adaptation that internationally qualified nurses undergo as they adjust to a new healthcare system.

Multiple international and Australian researchers have substantiated that internationally qualified nurses frequently feel professionally undervalued and underutilised, often being perceived as less competent despite their previous clinical experience (Deegan and Simkin, 2010; Kurniati et al., 2017; Smith et al., 2011). Such perceptions are further reinforced by workplace hierarchies that fail to recognise foreign qualifications and prior roles (Chun Tie et al., 2018; Kishi et al., 2014). Inadequate or inconsistent orientation programs have also been widely reported, with Internationally qualified nurses noting minimal institutional support, lack of role clarity, and insufficient mentoring during their early practice (Kurup et al., 2023; Tie et al., 2019). These systemic gaps exacerbate feelings of professional dissonance and contribute to a cycle of dissatisfaction and attrition (World Health Organization, 2020). Addressing these issues requires not only targeted transition frameworks but also critical examination of institutional biases and policy gaps that continue to hinder full integration.

The process of transition for internationally qualified nurses encompasses linguistic, cultural, and professional adaptation, all of which can be deeply challenging. Language barriers, especially among nurses from non-English-speaking backgrounds, are a significant issue, impeding effective communication with colleagues and patients (Philip, Woodward-Kron, et al., 2019; Timilsina Bhandari et al., 2015). Additionally, internationally qualified nurses often encounter cultural biases and discriminatory practices that further complicate their integration (Joseph et al., 2022). Professional challenges, such as being perceived as novices regardless of their expertise and skills, leave such many nurses feeling disempowered and professionally dissatisfied (Kurup et al., 2024; Vafeas and Hendricks, 2018). These issues are compounded by inconsistencies in the availability of structured orientation programs and support systems, which are crucial for fostering a smooth transition (Tie et al., 2019).

While prior researchers have explored aspects of these challenges, the literature is fragmented, and gaps remain in understanding the full scope of internationally qualified nurses’ experiences. In some studies, researchers have focused on isolated themes, such as language barriers (Philip, Woodward‐Kron, et al., 2019), workplace discrimination (Joseph et al., 2022), or credential recognition (Kurup et al., 2024), without integrating these dimensions into a comprehensive understanding of the transition experience. For instance, previous researchers have often focused narrowly on either language barriers or cultural adaptation without fully examining the interplay between these factors or their broader implications for job satisfaction and retention (Adebayo et al., 2021; Gao et al., 2020). Furthermore, there is limited exploration of how systemic issues, such as anti-discrimination policies and standardised skill recognition frameworks, can influence the integration of Internationally qualified nurses into the healthcare system (Zhong et al., 2023).

In this scoping review, we sought to explore what is known and identify potential knowledge gaps by systematically mapping and synthesising the literature on experiences of internationally qualified nurses transitioning into the Australian healthcare system. Employing the Patterns, Advances, Gaps, Evidence for practice and Research recommendations (PAGER) framework (Bradbury-Jones et al., 2022) enabled us to identify recurring patterns, highlight advancements, and pinpoint critical gaps, offering actionable evidence for practice and research recommendations. Understanding these experiences is vital for developing policies and interventions that can enhance internationally qualified nurses’ integration, job satisfaction, and retention, ultimately supporting a more inclusive and sustainable healthcare workforce in Australia.

## 2 Methods

We followed the five-step framework developed by Arksey and O’Malley (2005), which includes: (1) formulating research questions, (2) identifying relevant studies, (3) selecting eligible studies, (4) organising and charting the data, and (5) synthesising, summarising, and reporting the findings. In the final step, the PAGER framework (Bradbury-Jones et al., 2022) was applied to systematically analyse the results, ensuring the findings were actionable for practitioners, policymakers, and researchers. Reporting was guided by the PRISMA-ScR checklist (Tricco et al., 2018).

### 2.1 Identifying the research question

We aimed to explore and synthesise existing evidence on the transition experiences of internationally qualified nurses integrating into the Australian healthcare system. The main research questions were: (1) What are the experiences of internationally qualified nurses in transitioning into the Australian healthcare system? (2) How do internationally qualified nurses perceive and manage language and communication barriers in Australian healthcare settings? (3) What cultural and professional challenges do internationally qualified nurses face in integrating into the Australian healthcare workforce? (4) How are internationally qualified nurses’ professional identities and specialised skills perceived and recognised in Australian healthcare? (5) What types of support and resources do internationally qualified nurses find most beneficial in their journeys in becoming nurses in Australia?

### 2.2 Identification of relevant study

A literature search was conducted for studies published between January 2014 and December 2024 using the databases Ovid MEDLINE, EBSCO CINAHL, Embase, Scopus, and Web of Science Core Collections. To supplement this, grey literature was explored via the Google search engine, and reference lists of selected studies were reviewed for additional relevant sources. The search terms included phrases such as “foreign nurse*”, “migrant nurse*,” “Internationally qualified nurse*”, “internationally educated nurses*”, “transitioning”, “adapting,” “Australia,” “Australian healthcare,” “experience*,” “journey” and “migration”. Detailed information about the search strategy can be found in Supplementary Material Appendix 1.

### 2.3 Selection of the study

Inclusion and exclusion criteria were determined following the Population, Concept, and Context framework outlined by Peters et al. (2020). The target population comprised internationally qualified nurses who were either in the process of registration or had completed it to practise nursing in Australia. The concept focused on their experiences, challenges (such as regulatory hurdles, language barriers, and cultural differences), and facilitators or supports that assisted them, such as mentoring and training programs. The concept also explored strategies used to overcome these obstacles. Eligible studies were conducted in any Australian healthcare setting (e.g., hospitals, aged care, primary care), used any empirical methodology (qualitative, quantitative, or mixed methods), and available in full text. The context was the transition to becoming a registered nurse in Australia, including the requirements and regulations that must be navigated. The Covidence review manager was utilised to streamline the process of importing, organizing, removing duplicates, and screening all retrieved articles. Titles and abstracts were independently reviewed by two researchers to identify articles potentially meeting the inclusion criteria. Full texts of these selected articles were then assessed independently by the same two reviewers to determine eligibility for inclusion. Disagreements were resolved through discussion, and if consensus was not reached, a third reviewer adjudicated. Although Cohen’s kappa was not calculated, reviewer agreement was ensured through iterative consensus. Additionally, the reference lists of included articles were examined to locate any other relevant studies.

### 2.4 Charting the data

Key data from included studies were extracted and organised, covering aspects such as authorship, publication year, study location, research design, objectives, settings, participants’ country of origin, population demographics, sample size, and findings. Data extraction was performed independently by two reviewers, with any discrepancies resolved through discussion or by consulting a third reviewer when necessary.

### 2.5 Collating, summarizing, and reporting results

The PAGER framework was utilised to critically evaluate and describe the body of literature included in the review (Bradbury-Jones et al., 2022). Findings were organized according to several key dimensions according to the framework. Patterns, identified as key themes, were aligned with the research questions to provide structure. For each pattern, advancements in the field, whether theoretical or methodological, were highlighted, and areas of knowledge or practice requiring further exploration were noted. These insights formed the basis for providing contextualized evidence to inform practice and offering targeted research recommendations. In accordance with the objective of mapping the breadth of available evidence, we did not include a critical appraisal or risk of bias assessment (Peters et al., 2020).

## 3 Results

### 3.1 Identification of studies and study selection

**Fig. 1:**
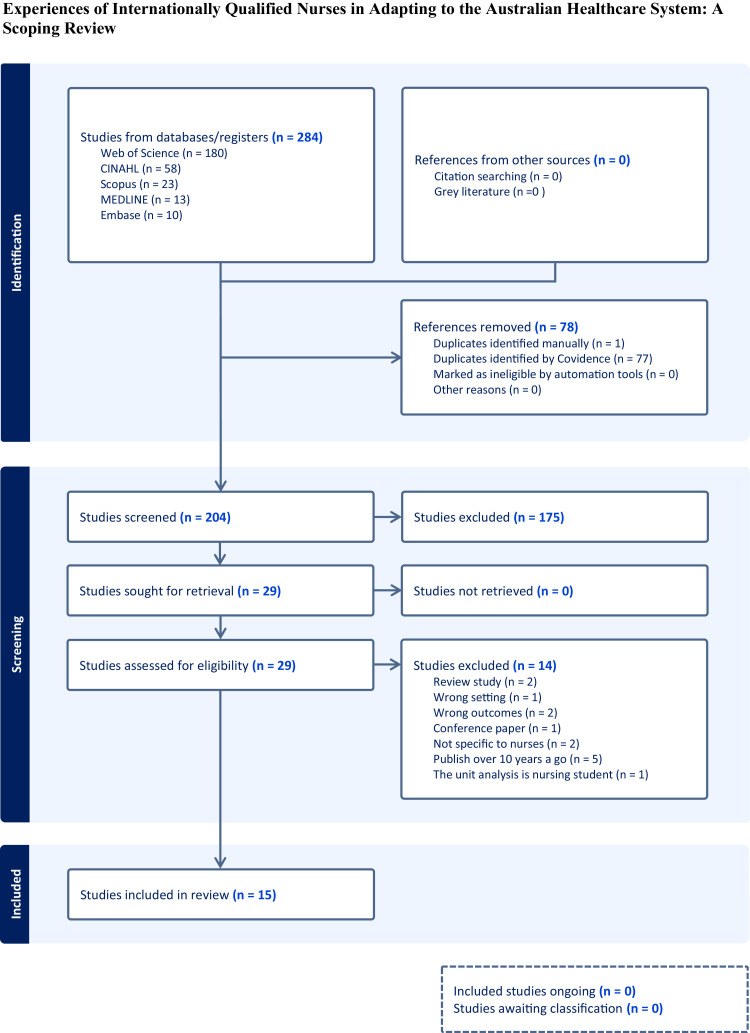
Experiences of internationally qualified nurses in adapting to the Australian healthcare system: a scoping review.

A total of 284 records were retrieved from the search. After eliminating 78 duplicates, 204 records proceeded to the title and abstract screening phase. Of these, 175 were excluded as they did not meet the inclusion criteria. A total of 29 studies were included for full-text review, of which 14 were excluded as they did not align with the study population. Ultimately, 15 studies met the eligibility criteria and were included in the final review (Fig. 1).

### 3.2 Characteristics of included studies

Of the 15 included studies, nine employed qualitative designs (Dywili et al., 2021; Gao et al., 2020; Joseph et al., 2022; Kishi et al., 2014; Philip, Woodward-Kron, et al., 2019, 2019; Tie et al., 2019; Vafeas and Hendricks, 2018; Zhong et al., 2023), four utilised cross-sectional designs (Adebayo et al., 2021; Kurup et al., 2024; Timilsina Bhandari et al., 2015; Zanjani et al., 2021), and two used mixed-methods designs (Aggar et al., 2020; O’Callaghan et al., 2018). All studies were conducted in Australia, with participants from various countries, including India, Philippines, China, Japan, sub-Saharan Africa, and the United Kingdom (UK). Study settings primarily included hospitals, residential aged care facilities, and specialised care units, with some participants drawn from bridging programs and mental health settings. Sample sizes of included studies varied between nine and 272 participants, totalling 943 individuals across all studies. Detailed characteristics of each study are presented in Table 1, while Table 2 presents the findings in relation to the five primary research questions. A summary of the PAGER framework analysis is provided in Table 3. The subsequent narrative synthesis presents the scoping review results, structured through insights derived from the framework.

**Table 1:** Overview of the characteristics of included studies.

Study ID	Country Origin	Aim	Study Design	Population Description	Sample Size	Key Findings
Adebayo et al., 2021	Sub-Saharan Africa, South Asian, Southeast Asian, Northeast Asian, others	To investigate acculturation stress impact on migrant care workers in aged care	Cross-sectional study	Migrant care workers in aged care facilities	272	High stress levels, skill underutilization common
Aggar et al., 2020	India, UK, China, Netherlands, Philippines	To investigate bridging program experiences for IQNs	Mixed methods study	IQNs in Graduate Certificate in Australian Nursing	9	Positive program experiences, autonomy and independence increased
Bhandari et al., 2015	India, China, Philippines, UK, 20 others	To explore job satisfaction factors among overseas-qualified nurses in South Australia	Cross-sectional study	Internationally qualified nurses in South Australian hospitals	151	Language, tenure, discrimination impact job satisfaction
Tie et al., 2019	Not specified	To explore adaptation process of international nurses in Australian healthcare	Qualitative research	International and Australian qualified registered nurses	217	Integration involves adapting to cultural norms, support enhances adaptation
Dywili et al., 2021	Sub-Saharan Africa	To explore migration experiences of sub-Saharan African nurses	Qualitative research	Sub-Saharan African RNs in rural NSW	18	Challenges of racial discrimination, coping strategies observed
Gao et al., 2020	Not specified	To explore lived experiences of international OR nurses in organ procurement	Qualitative research	International OR nurses in organ procurement	18	Cultural beliefs impact practice, support programs needed
Joseph et al., 2022	India	To explore transition experiences of Indian nurses in mental health	Qualitative research	Indian RNs in mental health settings	16	Cultural adjustment, discrimination challenges, eventual adaptation
Kishi et al., 2014	Japan	To investigate adaptation experiences of Japanese nurses in Australia	Qualitative research	Japanese registered nurses in Australian hospitals	14	Developed seeking, acclimatizing, and settling model for adaptation phases
Kurup et al., 2024	Not specified	To identify barriers and facilitators for specialty skill transfer	Cross-sectional study	IQNs with specialty skills in Australian healthcare	71	Skill recognition varies by state, transition support needed
O’Callaghan et al., 2018	Not specified	To explore support needs, attitudes in culturally diverse nursing environments	Mixed methods study	International and locally qualified nurses	108	IQNs face discrimination, language challenges, support needs differ by background
Philip S et al., 2019	Not specified	To explore interprofessional and intraprofessional communication patterns of OQNs	Qualitative observational study	OQNs in hospitals	13	Communication barriers in clinical settings, support for competency needed
Philip S et al., 2019	Philippines, India, Singapore, Africa	To explore barriers and enablers in clinical communication through community practice lens	Qualitative research	OQNs from NESB backgrounds	21	Disempowerment, support critical for effective practice
Vafeas and Hendricks, 2018	United Kingdom	To understand migration experience of RNs from the UK	Qualitative research	UK RNs in Western Australia	21	Employment challenges, professional identity affected
Zanjani et al., 2021	India, Philippines, China, others	To examine factors associated with sociocultural adaptation of OQNs	Cross-sectional study	OQNs working as registered nurses in Australia	200	Job satisfaction, supportive environment influence adaptation
Zhong et al., 2023	China	To illuminate professional adaptation experiences of Chinese migrant nurses	Qualitative research	Australian registered nurses of Chinese ethnicity	17	Adaptation involves self-evaluation, organizational support needed

Notes: IQNS=Internationally qualified Nurses; OQNs: Overseas Qualified Nurses; OR: Operating Room; NESB: Non-English Speaking Background; UKRNS: United Kingdom Registered Nurses.

**Table 2:** Summary of findings.

Study ID	Research Question 1: Experiences transitioning into Australian healthcare system	Research Question 2: Language and communication challenges	Research Question 3: Cultural and professional challenges	Research Question 4: Recognition of professional identity and skills	Research Question 5: Support and resources for Internationally qualified nurses
Adebayo et al., 2021	High acculturation stress affects adaptation in aged care settings	Language proficiency expected but transition still challenging due to medical terminology	Non-work-related stressors complicate adaptation	Mismatched qualifications and roles impact satisfaction	No specified support mechanisms for cultural and professional integration
Aggar et al., 2020	Positive experience in bridging program enhanced clinical confidence	–	Cultural differences in nursing practices affected adjustment	Increased autonomy in nursing roles noted	Bridging program and transition programs recommended
Bhandari et al., 2015	Decline in job satisfaction over time due to lack of recognition and integration	Language and communication barriers impact job satisfaction, especially for NESB nurses	Discrimination and feelings of 'otherness' lower morale	Skills underutilized; limited career advancement	Lack of structured support for adaptation
Tie et al., 2019	Integration requires adapting to Australian cultural norms in healthcare	Challenges with informal language and clinical jargon	Uncertainty about roles and cultural differences impact adaptation	Need for better understanding of role scope	Supportive colleagues and orientation programs crucial for adaptation
Dywili et al., 2021	Positive initial experiences, but racial discrimination impacts retention	–	Workplace discrimination challenges adaptation in rural settings	Cultural identity clashes impact professional integration	Developing coping strategies essential
Gao et al., 2020	Experiences in organ procurement require adjustment to different cultural views	Language and terminology barriers noted in specialized settings	Differences in cultural beliefs about organ donation impact professional integration	–	Support programs and coping skills identified as beneficial
Joseph et al., 2022	Mixed transition experiences with challenges balancing dual cultures		Discrimination and cultural clashes add stress	–	Long-term adaptation includes growth opportunities
Kishi et al., 2014	SAS model outlines stages of adaptation specific to Japanese nurses	Language issues with terminology during acclimatization phase	Maintaining cultural identity versus adapting to new environment	–	Supportive colleagues aided adaptation
Kurup et al., 2024	Transition facilitated by understanding scope of practice	–	Challenges due to variability in specialist skill recognition across states	Specialized skills underutilized	Tailored transition pathways needed
O’Callaghan et al., 2018	Opportunities and challenges in adapting to culturally diverse environments	Struggles with language use and professional interactions	Discrimination affects comfort and adjustment	Varied perspectives on support and recognition based on cultural background	Support resources recommended to ease transition
Philip S et al., 2019	–	Communication barriers impact comfort with English-speaking colleagues	Non-inclusive behavior affects psychological well-being	Need for more engagement with established team members	Mentorship and tailored support crucial
Philip S et al., 2019	–	Intraprofessional and patient communication challenging due to language issues	Feelings of disempowerment from communication difficulties	Reconsideration of professional identity necessary	Ongoing support and feedback aid in full participation
Vafeas and Hendricks, 2018	UK nurses experience professional dissonance in adaptation	–	Struggles in maintaining professional identity in new environment	Challenges in regaining respect and recognition	Belonging critical for successful adaptation
Zanjani et al., 2021	Higher adaptation linked to supportive work environments	Poor initial communication skills challenge integration	Cultural differences and job satisfaction impact adaptation	Sense of belonging enhances successful transition	Positive work environment and family connections aid integration
Zhong et al., 2023	Chinese migrant nurses experience duality in adapting professionally	Language barriers mixed with evolving self-assessment	Adapting involves retaining some cultural identity while adopting new norms	Self-evolution needed to match healthcare expectations	Both individual and organizational support essential for growth

Note: ID = Identifier; NESB = Non-English Speaking Background; SAS= Seeking Acclimatizing Settling.

**Table 3:** PAGER frameworks.

Pattern	Advance	Gap	Evidence for Practice	Research Recommendation
Language and Communication	Increasing use of bridging programs tailored for NESB nurses to support language adaptation	Insufficient language and communication support, especially for NESB nurses adapting to clinical jargon	Develop tailored language support programs for NESB nurses, especially focused on medical terminology	Conduct studies to assess the impact of targeted language support on NESB nurse retention and satisfaction
Discrimination and Cultural Challenges	Growing awareness of the need for inclusive practices and anti-discrimination policies in healthcare	Inconsistent application of anti-discrimination policies and lack of resources addressing workplace racism	Implement policies fostering inclusivity, reduce discrimination, and support cultural competence	Longitudinal studies tracking the impact of anti-discrimination policies on job satisfaction and retention
Professional Identity and Skills Recognition	Recognition of the need for standardized frameworks for skill validation of Internationally qualified nurses	Lack of clear pathways for recognizing Internationally qualified nurses' specialized skills and prior experience	Develop recognition pathways that support IQNs in utilizing their prior expertise fully	Research on the effectiveness of skill recognition programs in enhancing job satisfaction among IQNs
Support Systems and Adaptation	Emphasis on structured orientation and mentoring for smoother transitions	Inconsistent availability of orientation and support programs across institutions	Establish standardized orientation programs and mentorship initiatives for all institutions	Evaluate and compare the effectiveness of various support models to identify best practices

Note: PAGER= Patterns, Advances, Gaps, Evidence for practice and Research recommendations; IQNS=Internationally qualified Nurses; NESB: Non-English Speaking Background.

### 3.3 Experiences of internationally qualified nurses transitioning into the australian healthcare system

The experiences of internationally qualified nurses in Australia revealed a complex adaptation journey marked by both positive opportunities and significant challenges. Researchers have reported that, while internationally qualified nurses generally appreciated the structure and resources of the Australian healthcare system, adapting to new cultural and professional norms required substantial adjustment (Adebayo et al., 2021; Joseph et al., 2022; Kishi et al., 2014). O'Callaghan et al. (2018) and Tie et al. (2019) observed that many internationally qualified nurses found value in supportive colleagues and orientation programs, which helped bridge cultural differences and clarify workplace expectations (O’Callaghan et al., 2018; Tie et al., 2019). However, Vafeas and Hendricks (2018) and Kishi et al. (2014) highlighted a recurring theme of “professional dissonance”, where internationally qualified nurses often struggled to reconcile their prior expertise with the demands of a new system (Kishi et al., 2014; Vafeas and Hendricks, 2018). This dissonance impacted their sense of professional identity, as they were sometimes placed in roles that did not fully utilise their skills. Professional dissonance was seen to occur because internationally qualified nurses often faced under-recognition of their skills due to systemic mistrust of foreign qualifications and risk-averse employment practices. It manifested through limited responsibilities, exclusion from decision-making, and subtle discrimination, leading to emotional strain, loss of identity, and decreased job satisfaction (Kishi et al., 2014).

### 3.4 Language and communication challenges

Language and communication barriers emerged as a significant challenge for many internationally qualified nurses, especially those from non-English-speaking backgrounds. Two research teams identified that difficulties with accents, colloquial language, and specialised medical terminology created communication anxiety and could hinder effective practice (Philip, Woodward-Kron, et al., 2019; Timilsina Bhandari et al., 2015). These challenges were particularly pronounced in clinical settings, where effective communication is crucial for patient safety and interprofessional collaboration. Gao (2020) reported that Internationally qualified nurses often resorted to short, task-oriented interactions, which limited their social integration and affected job satisfaction (Gao et al., 2020). Philip, Woodward-Kron, et al. (2019) also noted that non-English-speaking backgrounds nurses often felt isolated due to these language difficulties.

### 3.5 Cultural and professional challenges in integration

Cultural differences and experiences of discrimination were prevalent challenges impacting internationally qualified nurses’ integration into the Australian healthcare system. Dywili et al. (2021) and Joseph et al. (2022) documented instances of racial discrimination and feelings of "otherness" (a sense of being excluded or treated as an outsider) in workplace interactions, which negatively impacted job satisfaction and retention (Dywili et al., 2021; Joseph et al., 2022). This was particularly problematic for nurses from regions with starkly different cultural backgrounds, where they may face bias from colleagues and patients. Additionally, a few studies also highlighted that internationally qualified nurses often encountered workplace practices and norms that differed from those in their home countries, creating additional adaptation barriers (O’Callaghan et al., 2018; Zanjani et al., 2021).

### 3.6 Recognition of professional identity and skills

Professional identity and skill recognition emerged as recurring themes in several studies. Many internationally qualified nurses reported feeling that their specialised skills and prior experience were not adequately acknowledged in Australian healthcare. Kurup et al. (2024) and Vafeas and Hendricks (2018) highlighted that nurses from the UK, India, and other regions experienced a “loss of autonomy” and frustration when they were unable to utilise their full skill sets. This gap between their expertise and the roles they were assigned contributed to a sense of underutilization and professional dissatisfaction (Kurup et al., 2024).

### 3.7 Support and resources beneficial for internationally qualified nurses’ integration

Support systems, such as orientation programs and mentorship, were key facilitators in the integration process for internationally qualified nurses. O'Callaghan et al. (2018); Aggar et al. (2020), and Tie et al. (2019) all underscored the importance of structured orientation programs that help Internationally qualified nurses understand local clinical practices and professional expectations (Aggar et al., 2020; O’Callaghan et al., 2018; Tie et al., 2019). Supportive colleagues and mentorship also played a crucial role, providing guidance that helped these nurses feel more included in the workplace. Researchers have suggested that Internationally qualified nurses benefit from peer support, which builds confidence and reduces transition stress (Tie et al., 2019).

## 4 Discussion

The experiences of internationally qualified nurses in Australia revealed a complex adaptation journey marked by both positive opportunities and significant challenges. Researchers reported that while internationally qualified nurses generally appreciated the structure and resources of the Australian healthcare system, adapting to new cultural and professional norms required substantial adjustment (Kishi et al., 2014; Zanjani et al., 2021). Some internationally qualified nurses experienced “professional dissonance” when placed in roles that did not fully leverage their previous expertise, impacting their sense of professional identity and satisfaction (Kishi et al., 2014; Vafeas and Hendricks, 2018). Similar experiences have been reported by Middle Eastern midwives transitioning into the Australian healthcare system, who described needing to reframe their professional identities to fit the new system (Safari et al., 2022). These individual experiences of professional dissonance and underutilisation among Internationally qualified nurses mirror the broader systemic challenges afflicting the global nursing workforce, where unmet needs and unacknowledged struggles continue to compromise retention, well-being, and workforce sustainability (ICN, 2025).

Emotional strain associated with adapting to new roles was compounded by discrimination from colleagues and patients (Dywili et al., 2021). Effective role-matching and recognition of prior skills could enhance the integration experience for internationally qualified nurses, underscoring the gap in policies supporting more tailored placement practices. We have echoed the findings of an International Council of Nurses 2025 global survey, which highlighted that nurses worldwide, particularly those who migrate, face increasing demands, under-recognition of their expertise, and frequent role mismatches that compromise their well-being and job satisfaction. The lack of national strategies for effective role recognition and placement was also noted as a critical gap in sustaining an equitable and resilient global nursing workforce (Sharplin et al., 2025).

Language and communication challenges emerged as critical patterns in the experiences of internationally qualified nurses, particularly those from non-English-speaking backgrounds. Communication issues have often arisen due to difficulties with accents, colloquial expressions, and specialised medical terminology, leading to anxiety and reduced social integration (Philip S et al., 2019; Timilsina Bhandari et al., 2015). Language barriers often limited interactions to task-focused exchanges, reducing social engagement and affecting job satisfaction (Gao et al., 2020). The evidence for practice suggests the need for healthcare organizations to implement targeted language-support programs, focusing on medical and colloquial language training, to enhance the confidence and competency in clinical communication of nurses from non-English-speaking backgrounds (Kurup et al., 2025; Philip, Woodward-Kron, et al., 2019). The latest International Council of Nurses global survey emphasised that nurses from non-English-speaking backgrounds often face systemic disadvantages in host countries, with limited language support identified as a key contributor to professional marginalisation and reduced well-being (Sharplin et al., 2025). Healthcare systems should implement structured, context-specific language support programs, integrated into onboarding and ongoing professional development to enhance communication confidence, reduce marginalisation, and improve retention of internationally qualified nurses from non-English-speaking backgrounds.

Discrimination and cultural bias were recurrent patterns across multiple studies. Instances of racial discrimination that lead to feelings of "otherness", negatively impacting job satisfaction and retention, were well-documented (Dywili et al., 2021; Joseph et al., 2022). Internationally qualified nurses, particularly those from non-English speaking backgrounds, often faced bias and felt socially isolated within their work environments (Adebayo et al., 2021). Discrimination occurs because internationally qualified nurses, especially from non-English speaking backgrounds, were often perceived as less competent due to language or cultural differences. This led to exclusion from clinical discussions and social networks, causing feelings of otherness, reduced job satisfaction, and higher turnover (Dywili et al., 2021). Addressing these biases requires organisational-level changes, including anti-discrimination policies, cultural competency training, and promoting inclusive environments. While some organisations are making initial progress in this area, as reflected in advances such as preliminary anti-discrimination policies, gaps remain inconsistent in application across institutions (O’Callaghan et al., 2018). Future efforts should, therefore, focus on the consistent and system-wide integration of mandatory anti-discrimination frameworks and culturally safe workplace practices to ensure equitable support and inclusion for internationally qualified nurses across all healthcare settings.

Limited recognition of internationally qualified nurses' skills and professional identities emerged as a significant pattern impacting job satisfaction and retention. Kurup et al. (2024) and Vafeas and Hendricks (2018) reported that internationally qualified nurses from various regions, such as the UK, India, and the Philippines, frequently encountered lost autonomy when their specialised skills were underutilized. Maeda and Socha-Dietrich (2021) highlighted that 79 % of nurses globally reported being over-skilled for their daily roles, with their expertise routinely underutilised, leading to widespread dissatisfaction. This mismatch between qualifications and assigned roles points to a gap in standardised frameworks for validating internationally qualified nurses’ expertise. Aligning their roles with their expertise could enhance professional identity and satisfaction, fostering a sense of empowerment and commitment to the healthcare system. The emerging recognition of the need for structured frameworks to facilitate the integration of internationally qualified nurses, evidenced by preliminary efforts in skill validation, represents a constructive development, yet highlights the persisting gap in comprehensive and systemic implementation (Booth et al., 2025). Future researchers should explore how standardised skill recognition frameworks and culturally responsive integration strategies influence internationally qualified nurses’ ability to practise autonomously and how these mechanisms affect long-term professional satisfaction and workforce retention.

Supportive resources, such as structured orientation programs and mentorship, were consistently identified as beneficial patterns in facilitating internationally qualified nurses' successful integration (Aggar et al., 2020; Tie et al., 2019). Orientation programs significantly reduce transition stress and promote a sense of belonging by helping internationally qualified nurses understand local practices, workplace expectations, and professional standards (Aggar et al., 2020; O’Callaghan et al., 2018). Mentorship and support from peers also provided social and emotional stability, helping internationally qualified nurses build confidence and adapt more smoothly (Tie et al., 2019; Zhong et al., 2023). While structured support is increasingly recognized as essential, gaps in the availability of these programs across institutions reveal the need for standardisation (Gillis, 2024). By establishing uniform orientation and mentorship models, healthcare organisations could enhance the integration experience for internationally qualified nurses, reduce transition stress, and promote a stronger sense of belonging. A recommendation for future researchers is to examine how variations in the design and delivery of orientation and mentorship programs across institutions influence internationally qualified nurses’ integration outcomes, to inform the development of consistent, evidence-based support models.

We have highlighted several practical and policy implications. Healthcare organisations should focus on developing culturally competent, inclusive workplaces where internationally qualified nurses feel supported and valued. Tailored language support, anti-discrimination initiatives, and skill recognition frameworks could greatly enhance their adaptation experience. Policymakers should also consider creating standardised support systems across healthcare institutions, ensuring that internationally qualified nurses have equitable access to resources regardless of where they work.

While several researchers reported perceived short-term benefits of orientation and language programs, such as easing initial transition and improving communication confidence, their studies were typically cross-sectional or qualitative with limited follow-up. Future researchers should therefore focus on evaluating the long-term effectiveness of these interventions, particularly regarding their sustained impact on job satisfaction, professional identity, and retention of internationally qualified nurses. Longitudinal studies tracking their adaptation over time would offer valuable insights to inform more responsive and enduring support strategies.

### 4.1 Limitations

This review was limited to English-language studies, potentially excluding relevant research published in other languages, particularly from countries where internationally qualified nurses working in Australia originate. Furthermore, the focus on literature published from 2014 onward may have excluded earlier studies that could have provided valuable historical insights. Despite these limitations, we have offered a comprehensive synthesis of recent literature on the experiences of internationally qualified nurses transitioning into the Australian healthcare system, covering nearly a decade of research from 2014 to August 2024. The inclusion of diverse study designs and perspectives further enhanced understandings of the challenges and facilitators in the integration process.

## 5 Conclusions

We have revealed a multifaceted transition journey marked by significant challenges in cultural adaptation, language and communication barriers, limited recognition of professional skills, and inconsistencies in support resources. These challenges underscore the need for enhanced support structures to facilitate internationally qualified nurses’ integration and professional development. Several key gaps were identified in current support systems, including the need for tailored language programs, consistent recognition of internationally qualified nurses’ qualifications, and implementation of standardised orientation and mentorship programs. Workplace discrimination and cultural biases were frequently reported, pointing to the importance of inclusive practices and anti-discrimination policies within healthcare settings. Addressing these gaps may be crucial for improving job satisfaction, retention, and overall integration experiences of internationally qualified nurses in Australia. Ongoing research and policy development are essential to support the professional growth of internationally qualified nurses and ensure the Australian healthcare workforce can effectively integrate and retain skilled international professionals.

## Funding

This study is funded by Universitas Airlangga under the Penelitian Unggulan Airlangga research scheme, grant number 363/UN3.LPPM/PT.01.03/2024.

## CRediT authorship contribution statement

**Ferry Efendi:** Writing – review & editing, Supervision, Methodology, Funding acquisition, Formal analysis, Data curation, Conceptualization. **Rifky Octavia Pradipta:** Writing – review & editing, Methodology, Formal analysis, Conceptualization. **Makhfudli Makhfudli:** Writing – review & editing, Investigation, Conceptualization. **Lisa McKenna:** Writing – review & editing, Validation, Supervision. **Grace Solely Houghty:** Writing – review & editing, Validation, Supervision. **Fitri Kurnia Rahayu:** Writing – original draft, Methodology, Formal analysis. **Fildzah Cindra Yunita:** Writing – original draft, Methodology, Investigation.

## Declaration of competing interest

The authors declare that they have no known competing financial interests or personal relationships that could have appeared to influence the work reported in this paper.

## Appendix Supplementary materials

Image, application 1
