# Supplementary material for: Experiences of internationally qualified nurses in adapting to the Australian healthcare system: A scoping review
Source: Int J Nurs Stud Adv. 2025 Aug 5;9:100399. doi: 10.1016/j.ijnsa.2025.100399 (PMC12359234; doi:10.1016/j.ijnsa.2025.100399)
Supplement: Supplementary file 1 [file mmc1.docx]

**Appendix: Detailed Search Strategy**

To ensure a comprehensive and reproducible literature search, a systematic strategy was developed and implemented across five electronic databases: **Ovid MEDLINE**, **EBSCO CINAHL**, **Embase**, **Scopus**, and **Web of Science Core Collection**. The search covered literature published between **January 2014 and August 2024**. It included controlled vocabulary (e.g., MeSH terms) and free-text keywords, using Boolean operators (AND/OR) for combination across four conceptual domains.

| Concept | Keywords |
| --- | --- |
| Internationally Qualified Nurses | foreign nurse*, internationally qualified nurse*, overseas qualified nurse*, internationally educated nurses, migrant nurse*, immigrant nurse* |
| Transitional Processes and Experiences | transition*, adapting OR adaptation, integration, professional identity, role recognition, credential recognition, lived experience*, perception*, journey, narrative |
| Barriers and Challenges | language barrier*, communication challenge*, cultural competence, discrimination, workplace racism, role dissonance, skill underutilization |
| Context—Australian Healthcare System | Australia, Australian healthcare, Australian nursing workforce, health system, clinical practice environment |

**Inclusion Criteria**

- Empirical studies (qualitative, quantitative, or mixed methods)
- Published between 2014–2024
- Focus on internationally qualified nurses transitioning and adapting into the **Australian** healthcare system
- Reports on personal, professional, linguistic, or systemic adaptation experiences
- Peer-reviewed and published in English

**Exclusion Criteria**

- Studies focusing solely on Australian-educated nurses
- Theoretical papers, editorials, commentaries, or dissertations
- Non-English publications

**Supplementary Search Methods**

- **Grey literature search** using Google search engine
